# Dimensional Accuracy of Different Three-Dimensional Printing Models as a Function of Varying the Printing Parameters

**DOI:** 10.3390/ma17143616

**Published:** 2024-07-22

**Authors:** Christin Arnold, Lea Riß, Jeremias Hey, Ramona Schweyen

**Affiliations:** Department of Prosthodontics, Martin-Luther-University Halle-Wittenberg, Magdeburger Str. 16, 06112 Halle, Germany; christin.arnold@uk-halle.de (C.A.);

**Keywords:** 3D printing, dimensional accuracy, support structure, dental model

## Abstract

Even in digital workflows, models are required for fitting during the fabrication of dental prostheses. This study examined the influence of different parameters on the dimensional accuracy of three-dimensionally printed models. A stereolithographic data record was generated from a master model (SOLL). With digital light processing (DLP) and stereolithography (SLA) printing systems, 126 models were produced in several printing runs—SolFlex350 (S) (DLP, *n* = 24), CaraPrint 4.0 (C) (DLP, *n* = 48) and Form2 (F) (SLA, *n* = 54)—and their accuracy was compared with plaster and milled polyurethane models. In addition to the positioning on the build platform, a distinction was made between parallel and across arrangement of the models to the printer’s front, solid and hollow models, and printing with and without support structures. For accuracy assessment, five measurement sections were defined on the model (A–E) and measured using a calibrated digital calliper and digital scans in combination with the GOM Inspect Professional software 2021. The mean deviation between the measurement methods for all distances was 79 µm. The mean deviation of the models from the digital SOLL model were 207.1 µm for the S series, 25.1 µm for the C series and 141.8 µm for the F series. While positioning did not have an influence, there were clinically relevant differences mainly regarding the choice of printer, but also individually in alignment, model structure and support structures.

## 1. Introduction

The three-dimensional (3D)-printing process has become established and is frequently used, especially in the production of dental models [1,2,3,4]. Consequently, not least because of the material-efficient additive production, many manufacturers have included a 3D printer in their portfolio [3]. Because it is a comparatively inexpensive purchase, printers with stereolithography (SLA) or digital light processing (DLP) technology are increasingly used. In both technologies, the printers contain a vat filled with synthetic resin. During the printing process, a build platform is pulled out of the vat step by step. In this process, a laser beam (SLA principle) or a projected image (DLP principle) polymerises successively layer by layer through the bottom of the printer. In SLA printers, a laser beam is directed to the bottom of the vat using mirror galvanometers in a Cartesian coordinate system to polymerise the resin point by point [5,6]. The arrangement of the laser and the mirrors is specific for each printer. Depending on the build platform, the illuminated mask varies in DLP technology. Micromirror devices with a high-power light source are used [7]. The layer-building process is repeated in both methods until the object is completely formed. Freshly printed objects must be post-processed to achieve their maximum mechanical stability [8].

Several studies have shown that printing parameters, including the user-selected position of the models on the build platform, whether the models are solid or hollow and the layer thickness, influence the results [9,10,11]. Solid models have dominated most of the published investigations; in comparison, hollow shell models are used less frequently [10,11]. Depending on the model and the positioning, support structures are required for stabilisation; they have an influence on the surface roughness depending on the angle of inclination [12].

Dental models must meet different dimension specifications. The clinically acceptable values of accuracy vary greatly in the relevant literature. For example, an accuracy of less than ±200 to even less than 500 µm is postulated for planning and situation impressions [1,13,14], and no more than ±100 µm for master models or saw-cut models, which are used for the production of fixed partial dentures such as crowns, bridges and implant-supported dentures [10,15]. Gypsum is the conventionally used analogous material for the fabrication of dental models. Its accuracy can be fixed at a value of less than ±50 µm and even ±10 µm with the use of an appropriate manufacturing process and additives (resin that is reinforced or mixed with epoxy resin). These measurements are at defined distances and taken 1 day after casting [16]. The resin components, which are processed with 3D printers with SLA or DLP technology, are rather contractive during the polymerisation or curing processes [3,17,18].

The available data for determining the dimensional accuracy of models are mostly limited to fully anatomic jaws that contain all teeth [5,19,20,21,22,23,24]. In rare cases, researchers have investigated a jaw quadrant or the area that will be prosthetically restored, as is common in practice [17,25,26,27,28]. Potentially complicated wide-spanning dental arches are also considered to be potentially more prone to complications, solely due to the scanning process [26,29]. In addition, the focus of the comparison is often on the scanner that is used [30,31], and less frequently on the accuracy of the printers [3,24,32,33].

The primary purpose of this in vitro study was to determine the dimensional accuracy of models of fixed dental prostheses compared with a master model as a function of printer selection, positioning and placement on the build platform and the model shape (hollow vs. solid). The following four hypotheses related to the primary purpose were tested: (1) the use of different printers has no effect on the dimensional accuracy of the printed models; (2) the positioning or placement of the models on the respective build platform does not affect the dimensional accuracy; (3) there are no dimensional differences between solid and hollow models; and (4) there are no differences between models printed with and without support. The secondary purpose was to compare different measurement methods. In the relevant literature, measurements with callipers [11,22,34,35] are represented as digital measurements by various software programs [5,19,22,24,35,36]. This secondary purpose tested hypothesis (5): the use of different measuring methods—calliper versus software—does not result in significantly different measurements with regard to the dimensional accuracy of the printed models.

## 2. Materials and Methods

### 2.1. Master Model

**Figure 1:**
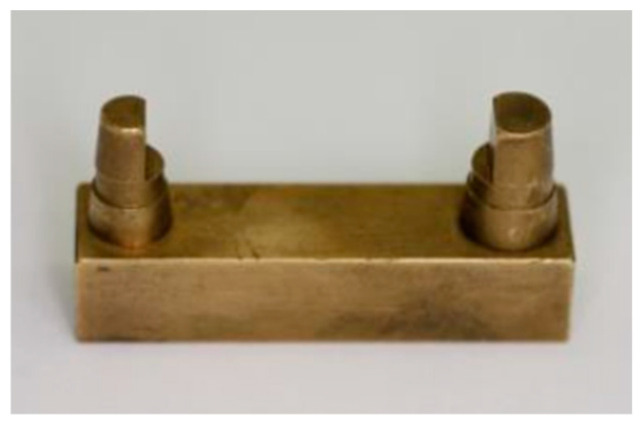
The master model.

**Figure 2:**
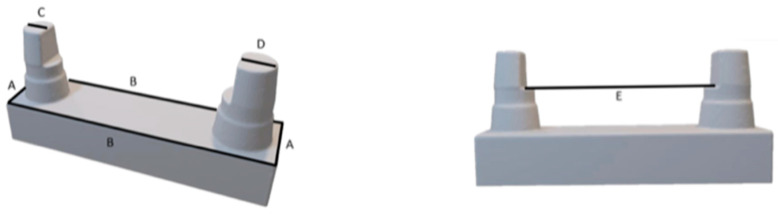
The measuring distances. Letters are described in Table 1.

The measurement was based on a proven master model made of brass (Figure 1) [12,37]. It simulates a clinical situation of a single-span, four-unit bridge. The small stump represents a canine, and the large stump represents a molar. Figure 2 and Table 1 indicate the distances that were measured for the comparison of dimensional stability.

To generate a digital data record according to the clinical situation, the master cast was coated with scan spray (3D Anti-Glare Spray, Organical CAD/CAM GmbH, Berlin, Germany) and then scanned with the highest detail level for the stump and arc scan with a scanner (D2000, 3Shape, Copenhagen, Denmark). The resulting stereolithographic (stl) data record was the basis for the additively manufactured solid models. The Meshmixer 3D modelling program (Autodesk Research, Toronto, ON, Canada) was used to create hollow models.

### 2.2. 3D Printing of the Test Models

Three dental 3D printers with two different functional mechanisms were selected for the additive manufacturing of the models. Table 2 presents an overview of the printers and the printing materials.

**Figure 3:**
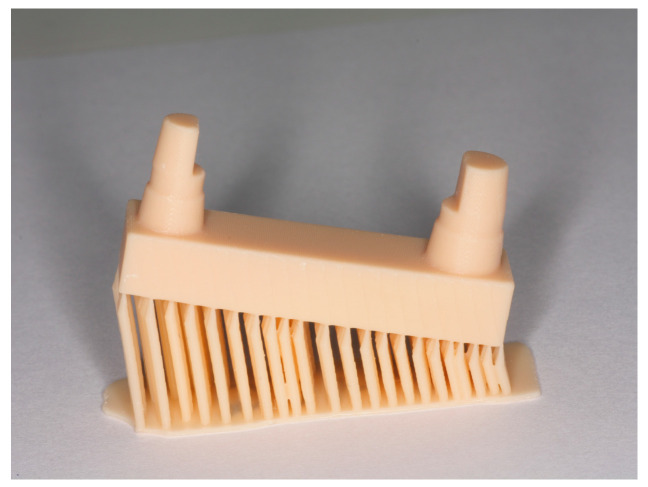
Support structure of the C series.

**Figure 4:**
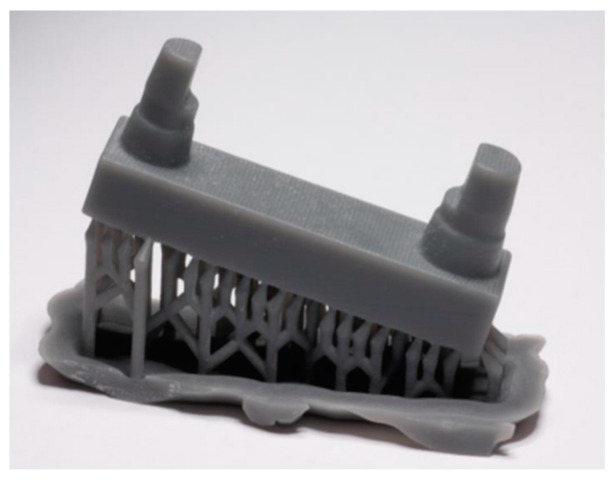
Support structure of the F series.

To ensure comparability, at least one match was made for each printing parameter and for each printer (Table 2). The 15° inclination of the models is shown in Figure 3 and Figure 4. The assembled canine stump is in an elevated position.

**Figure 5:**
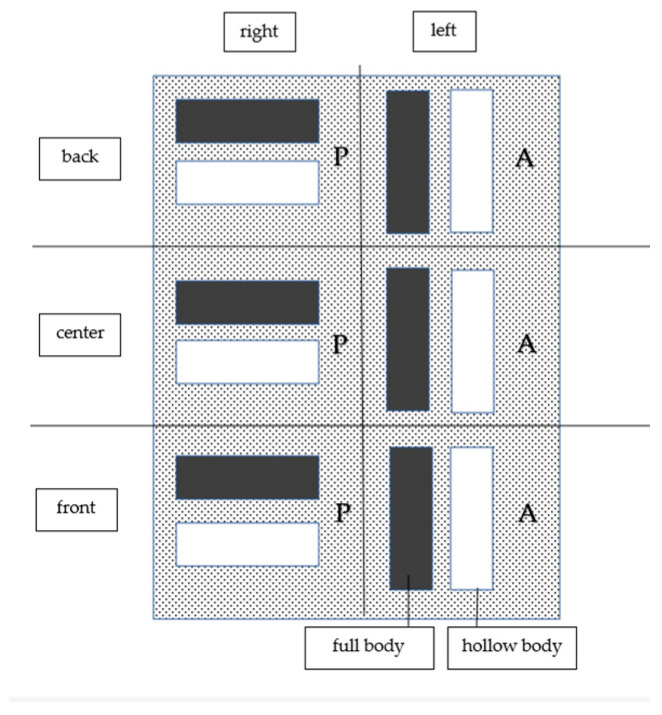
S-series models on the build platform. P = parallel, A = across.

**Figure 6:**
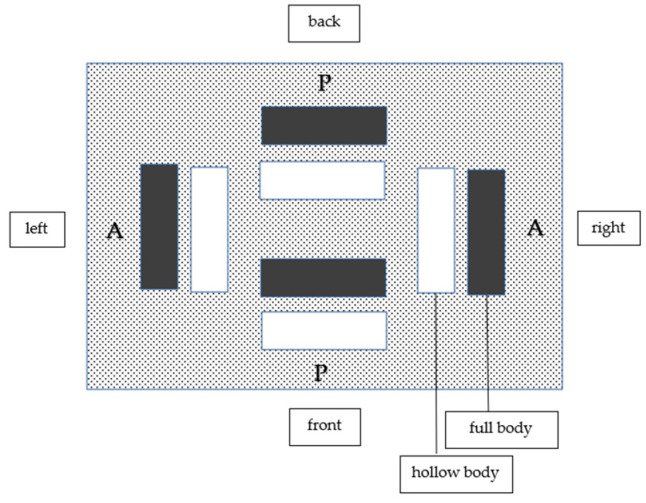
C-series models on the build platform. P = parallel, A = across.

**Figure 7:**
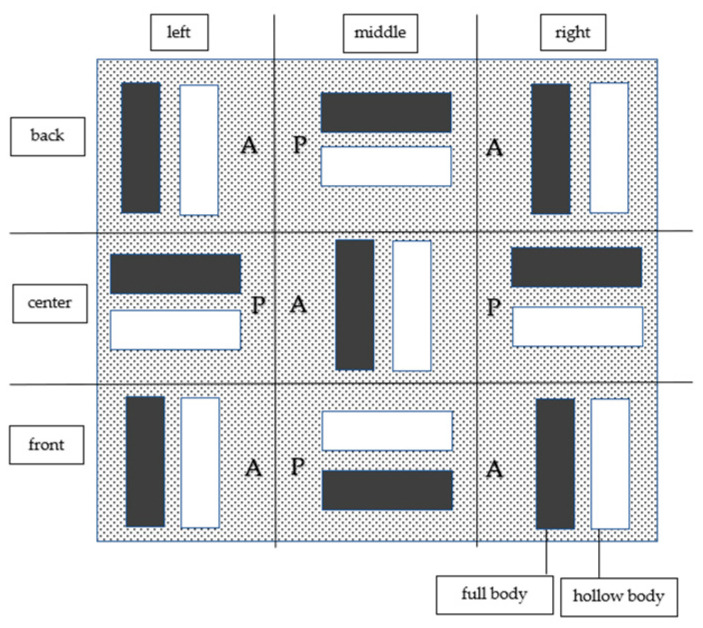
F-series models on the build platform. P = parallel, A = across.

Altogether, 126 models were produced, of which 24 were produced by the Solflex 360 (the S series), 48 by the CaraPrint 4.0 printer (the C series) and 54 by the Form 2 printer (the F series). The different number of models for each series is due to the morphology of each printer’s build platform (Figure 5, Figure 6 and Figure 7, Table 2). Similarly to the pilot study and the power analysis of Anadioti et al., a minimum of 20 models per test series were generated. In addition, several studies have used a minimum sample number of 10 models to test the influence of parameters [19,20,33,36,38]. Manufacturer- or printer-specific recommended resins were used for the models; their compositions are shown in Table 3.

### 2.3. Specificities of the Printing Processes and Post-Processing of the 3D-Printed Models

After printing, the models were post-processed according to the respective manufacturer’s specifications. Until the time of the measurements, the models were stored in a climate chamber at a constant temperature of 21 °C.

#### 2.3.1. S-Series Models and Post-Processing

After a 10 min draining period, the models were removed from the build platform and precleaned by repeated immersion in 98% pure isopropanol. Then, the models were placed in an ultrasonic bath (Sonorex RK100H, Badelin, Berlin, Germany) for 3 min. For the final cleaning, the models were placed in a fresh ultrasonic bath for 2 min. The models were dried with compressed air and placed in a xenon flashlight unit (Otoflash G171, dentona, Dortmund, Germany) for post-exposure, 15 min after the last isopropanol contact. The models were post-polymerised with two rounds of 2000 flashes (100 flashes per second), with a 2 min cooling phase between the rounds.

#### 2.3.2. C-Series Models and Post-Processing

After removing adherent liquid residue with compressed air, the models were removed from the build platform. The models were precleaned in an ultrasonic bath with 98% pure isopropanol for 3 min, and then post-cleaned in a fresh ultrasonic bath for 2 min. They did not spend more than 5 min in the ultrasonic bath. After cleaning, the models were dried with compressed air. The models were placed in a xenon flashlight unit (HiLite Power 3D, Kulzer GmbH, Hanau, Germany) two times at 5 min each: one time on the upper side and one on the lower side. Finally, the support structures were removed with a scalpel (HS Disposable Scalpel, Henry Schein Dental Deutschland GmbH, Langen, Germany).

#### 2.3.3. F-Series Models and Post-Processing

The models were removed from the printer and adherent liquid residue was rinsed off with 99.9% pure isopropanol. The support structures were removed with a mill (H251E.104.040, Gebr. Basseler GmbH & Co. KG, Lemgo, Germany). Subsequently, the models were cleaned two times (10 min each) with isopropanol on a vibration unit (KaVo EWL 5442, KaVo Elektrotechnisches Werk GmbH, Leutkirchen, Germany). Between the two cleanings, the released ingredients were removed with 3 bar of compressed air. The models were dried for 1 h at 60 °C in the drying oven (KaVo EWL TYP 5615, KaVo, Elektrotechnisches Werk GmbH). Then, they were post-polymerised for 60 min at 350–500 nm (LUXOMAT D, UVA and blue light tube with 350–500 nm wavelength, al dente Dentalprodukte GmbH, Horgenzell, Germany).

### 2.4. Reference Models

For reference, milled (Organical Multi Changer 20, OCAD CAM GmbH) polyurethane models (Organic Model blank, Organical CAD CAM GmbH) were fabricated using the *stl* dataset. For the plaster reference models, the master model was moulded with alginate (Tetrachrom, Kaniedenta GmbH & Co. AG, Herford, Germany) using an individual impression carrier and, following the manufacturer’s recommendations, cast with super-hard stone (Original Rocky Mountain [IV], Dental GmbH, Augsburg, Germany) immediately after carefully rinsing the impression with water and transferred into super-hard stone according to the manufacturer’s specifications.

### 2.5. Calliper Measurements

To evaluate the dimensional accuracy, the master, reference and printed models were measured under standardised conditions using a certified and calibrated calliper from Mitutoyo (ABS DIGIMA-TIC, Mitutoyo Europe GmbH, Neuss, Germany). The room temperature during the measurement was constant at 22 °C. The same investigator performed all of the measurements to keep the measurement error small. All distances listed in Table 1 and shown in Figure 2 were measured. The model was clamped between the straight surfaces of the calliper sectors to measure distances A, B, C and D. The distance between the stumps (distance E) was measured by opening the calliper until it was flush against the straight surfaces of the stumps. Each measurement was repeated five times. The mean value per section was calculated for each sample and transferred to an Excel file.

### 2.6. Digital Measurement

#### 2.6.1. Creation of a Test Specimen Scan Dataset

For the scanning process, the models were coated with a thin layer of scan spray (3D Laser Scanning Anti-Reflection Spray MATT, HELLING GmbH, Heidgraben, Germany) from a distance of 25 cm and then scanned with a desktop scanner (D2000, 3Shape) with the highest detail level for the stump and arc. For this purpose, the models were fixed on the platform of the scanner using plasticine (Blu Tack Scan Fix, Ivoclar Vivadent GmbH, Ellwangen, Germany). The scan data were saved in the *stl* format.

#### 2.6.2. Comparative Measurements with the GOM Inspect Professional Software

**Figure 8:**
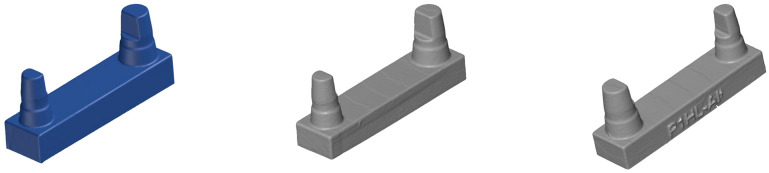
The SOLL model (left) and test models (IST; middle and right, lateral views) in the GOM Inspect Professional software 2021 before matching.

The matching process was performed using the GOM Inspect Professional 2021 software (Carl Zeiss GOM Metrology GmbH, Braunschweig, Germany). For this purpose, the .stl files were transferred to the software (Figure 8). In the software, the models were cut along the base plane in the *X*-axis to remove the plasticine base, which was necessary for scanning.

**Figure 9:**
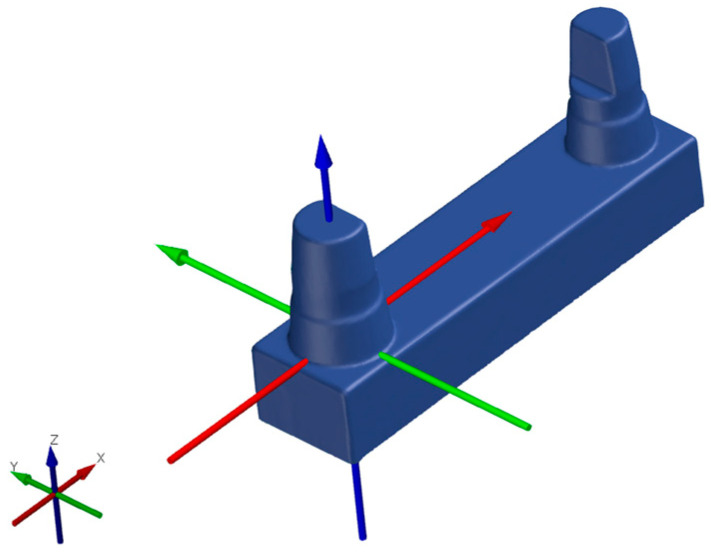
Alignment of the SOLL model in the 3D-coordinate system.

The scan data record of the master model was defined as the target model (SOLL model), while the printed models represented the IST models. To compare the SOLL model to all IST models, the SOLL model was treated as a pseudo-CAD. For correct and geometric positioning of the SOLL model in the 3D coordinate system of the GOM Inspect Professional software 2021, the scan data record of the SOLL model was subjected to a single-element transformation by auxiliary geometries (plane, line and point). Thus, the SOLL model could be moved in the global space and integrated in the coordinate system in an exactly defined way (Figure 9).

**Figure 10:**
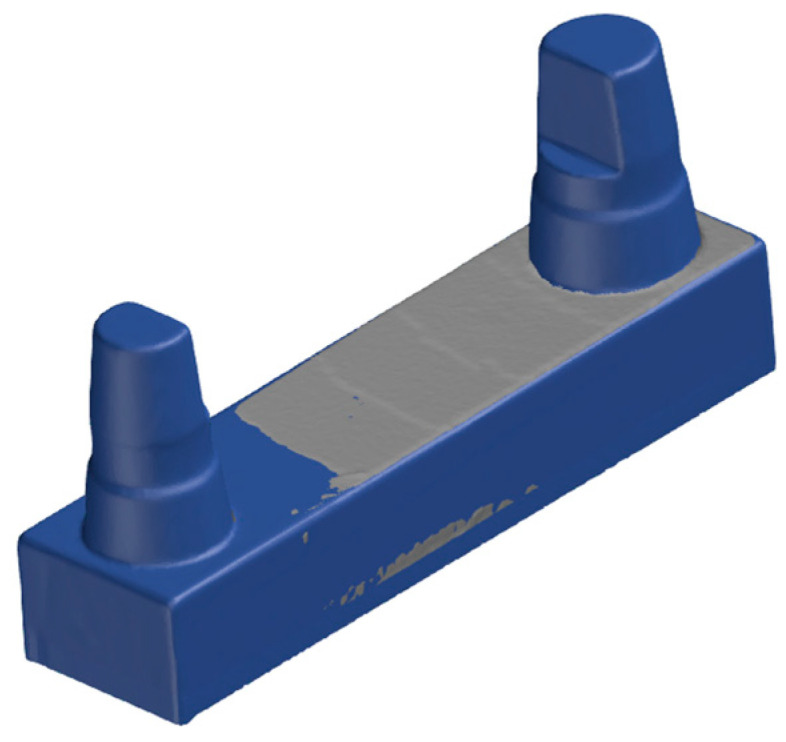
Initial alignment.

**Figure 11:**
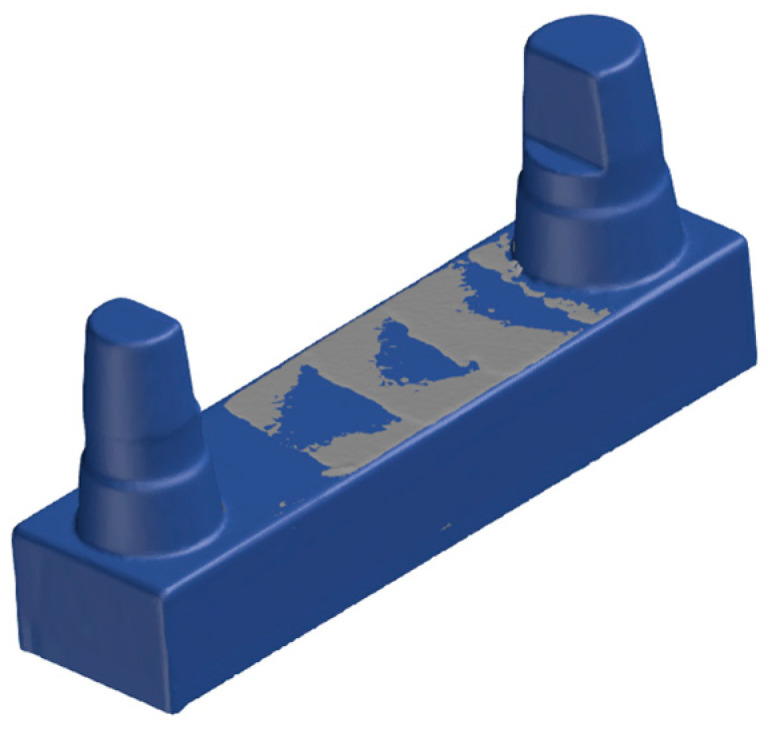
Main alignment.

**Figure 12:**
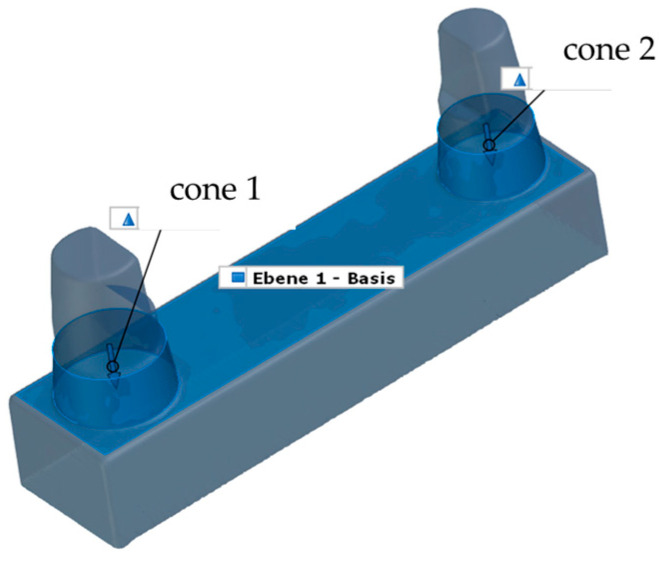
The geometric elements of the main alignment.

**Figure 13:**
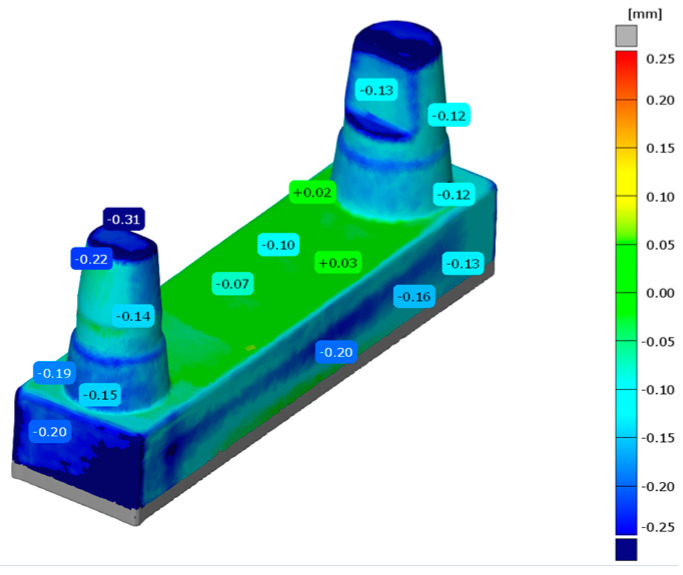
Area comparison of the master model and the printed models.

Using the automatic initial alignment function of the GOM Inspect Professional software 2021, the printed and reference models were initially aligned to the SOLL model (Figure 10). For a more accurate match, a main alignment was performed by using geometric elements (Figure 11). Thus, the models were aligned along the plane between the stumps and the two cones around the stumps (Figure 12). The deviations of the superimposed master model and printed model could be displayed in colour as an area comparison (Figure 13).

**Figure 14:**
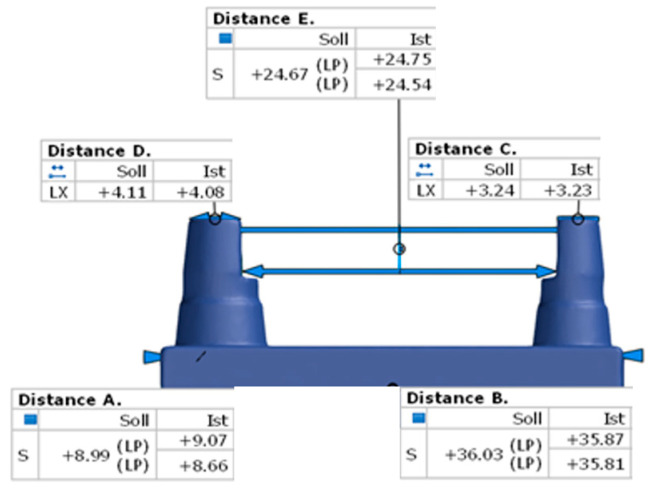
An example of the distance values between the master model and the test body.

A digital measuring method was used to compare the calliper measurements with the digital matching process. The selected measurement technology is frequently used in the automotive and aerospace industries but has also been used in the dental sector [21,32]. With the GOM Inspect Professional software, the distance between opposing planes was determined, an approach that resembles the calliper principle. To measure the models according to the independence principle, symmetry planes were defined on the parallel faces, and theoretical edge points of the stumps were determined in the two-dimensional (2D) view of the software. This measurement key was transferred from the SOLL model to all IST models to determine the distances between the planes and lines for each model. Because the 3D-printed model did not have a flat surface over the entire side, the software specified a minimum and maximum value for each model (Figure 14). From this, the average value was calculated (the largest area of triangles). The labelling on the models had no influence because all deviations > 5° from the surface were excluded from the digital measurement. The same investigator performed all of the digital and calliper measurements.

The mean values of each model were summarised in a table and transferred to Excel. The values of the SOLL model are shown in Table 4.

### 2.7. Evaluation and Statistical Analysis

Each model was coded based on its printing parameters. This allowed rating the various distances individually for each parameter. 

According to ISO 5725-1:2023, accuracy is divided into trueness and precision, which are quantitative counterparts. Accuracy is the degree of agreement between the arithmetic mean of a large number of test results and the true or accepted value. Precision is defined as the degree of agreement between test results and thus corresponds to the value of deviation with repeated measurement (repeatability value) [39]. 

To compare the measurement methods for each printer, the mean calliper and GOM Inspect Professional software measurements per distance were set in relation to the SOLL model. The statistical analysis was performed using SPSS Statistics version 21 (IBM corp. Armonk, NY, USA). Apart from descriptive statistics, the Kolmogorov–Smirnov and Shapiro–Wilk tests were used to check whether the data had a normal distribution. Levene’s test was used to assess the homogeneity of variances. For the normally distributed data, statistical differences were assessed with Student’s *t*-test or a univariate analysis of variance (ANOVA) followed by the post hoc Bonferroni test. The non-normally distributed data were assessed with the Mann–Whitney U test or the Kruskal–Wallis test. A *p*-value < 0.05 was considered to be statistically significant. 

## 3. Results 

### 3.1. Reliability

The accuracy of the measurement methods was investigated by assessing trueness and precision. Assessment of trueness requires the absolute value of the SOLL model; it can only be determined by using a higher-level measurement system (e.g., an Artos scanner) under known material conditions. This is not possible in a real patient case, so the true value of the model was not used. To assess the precision of the calliper measurements, the method error was evaluated according to Dahlberg’s method [40]. For this purpose, five samples from a printer were repeatedly measured with the calliper. There was sufficient measurement accuracy (0.047). To determine the precision of the GOM Inspect Professional software, 30 identical scans of the SOLL model were performed. The generated data record was matched with the SOLL model and measured. The average difference was 0.0101 mm, which is within the tolerance range. Thus, the measurement system itself did not generate fluctuations in the measurement results.

### 3.2. Comparison of the Measurement Methods

The SOLL and printed models were measured with a calliper and with the GOM Inspect Professional software. The calliper’s manufacturer specifies that its accuracy is ±0.02 mm. When measuring the SOLL model, there were clinically irrelevant differences of up to 10 µm for three distances (A, B and C; Table 5). 

To compare the measurement results between the methods, the mean value of all models of the respective measurement series was calculated for each defined distance. The basis for the comparison of the models was the measured values of the SOLL model in the GOM Inspect Professional software. All differences were determined for these results. For all but 3 of the 15 possible comparison cases, the differences between the calliper measurements and the SOLL model measurements were lower (Table 5, values in italics) and corresponded more to the SOLL model. Overall, the mean measurement deviation between the methods was 77 µm. The Kolmogorov–Smirnov test showed that the S- and C-series results were normally distributed, while the F-series results were not. A comparison of the measurement methods showed no relevant difference for the normally distributed data (*p* > 0.05). Assuming that a 3D measurement by means of geometric, flat elements produces more exact values and to exclude an improvement of the results, the statistical evaluation was based on the scanned models using the GOM Inspect Professional software (Table 5, values in bold).

### 3.3. General Results Compared with the Reference Models

As shown in Table 6, the GOM Inspect Professional software results for the printed models showed shorter distances compared with the digital SOLL model, so the real models were comparably smaller. The exceptions were distance E in the S series (+113 µm) and distance C in the C series (+20 µm). The C-series models showed the smallest differences (a maximum of 47 µm for distance B and a minimum of 6 µm for distance D) and greater agreement with the SOLL model than the classic plaster models (a maximum of 76 µm for distance A and a minimum of 22 µm for distance D), which were slightly enlarged. The greatest differences were in the S series (a maximum of 327 µm for distance B and a minimum of 112 µm for distance E). The milled plastic models were also smaller than the SOLL model, with only distances A and B showing differences of >100 µm (Table 6).

### 3.4. Comparison of the Results in Relation to the Printer Parameters 

#### 3.4.1. Model Structure: Hollow Versus Solid Comparison

**Figure 15:**
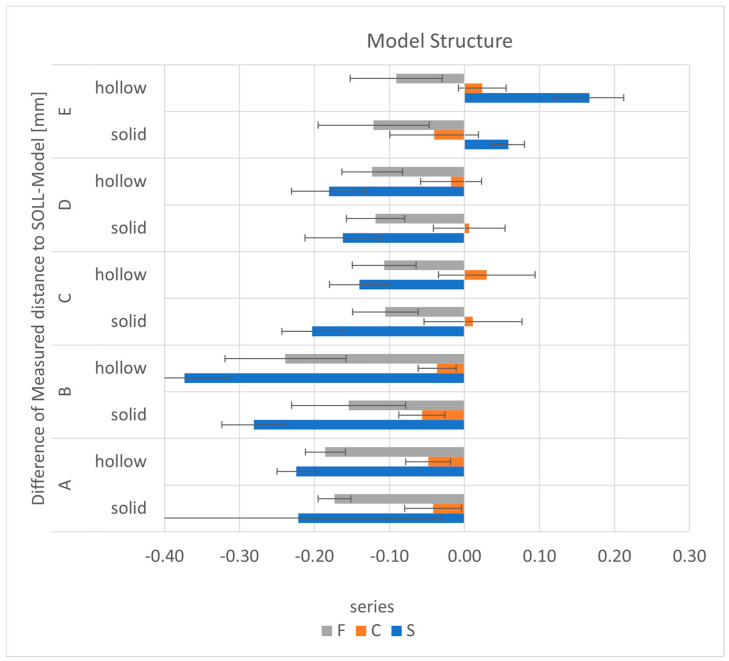
The mean differences as a function of the distances and the model structure for each series.

There were no uniform changes distributed over the measured distances (Figure 15). The S series showed a smaller difference for the hollow models at distance C (*p* < 0.002). At distance B (*p* < 0.001), but also minimally at distances A and D, the differences were smaller for the solid models. There was an evident extension for distance E, which was smaller for the solid models (58 µm) than for the hollow models (167 µm; *p* = 0.000). For the C series, the mean dimensional differences between the solid and hollow models did not exceed 20 µm and were both contractive and expansive. The difference in distance E (*p* < 0.000) and distance B (*p* < 0.02) was in favour of the hollow models. The F-series models were consistently contracted; there were no relevant differences between the solid and hollow models (a maximum of 30 µm) for distances A, C, D and E. There was a tendency for the differences to be smaller for the solid models, especially for distance B (*p* < 0.001). The only exception was distance E, although this was not statistically significant (*p* > 0.05).

#### 3.4.2. Model Base Orientation 

**Figure 16:**
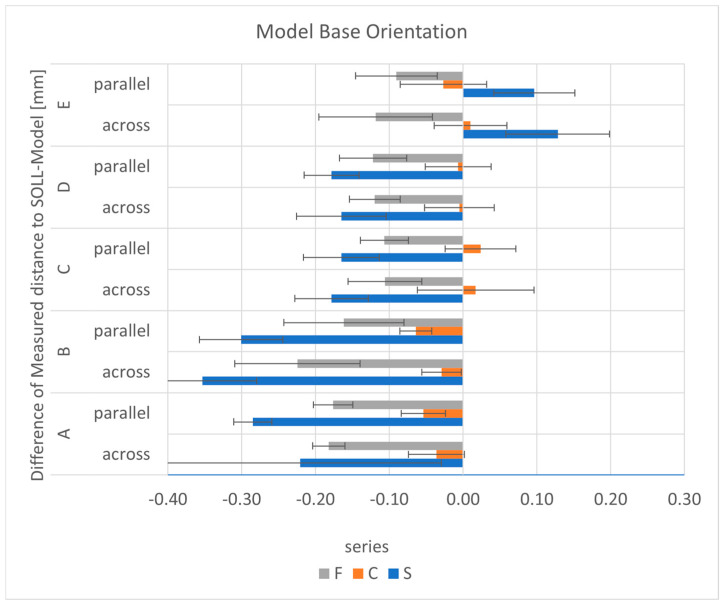
The mean differences as a function of the distances and the placement for each series.

In the S series, there were no differences between the across and parallel printed models in directions A, C and D (a maximum difference of 16 µm, Figure 16). For distances B and E, the parallel models were closer to the SOLL model. In contrast to the other distances, distance E showed expansion. Due to the large scatter, no difference was statistically significant (*p* > 0.05). The C-series models showed a maximum difference of 35 µm at distance B. At distances A, C, D and E, the differences between the parallel and across orientations were <17.5 µm and both expansive and contractive. The differences for the across printed models tended to be closer to the SOLL model at distances B (*p* < 0.000) and E (*p* < 0.026). For the F series, the largest difference between the across and parallel models was for distance B (63 µm, *p* < 0.000). For distances B and E, the parallel models showed smaller differences relative to the SOLL model. The differences for distances A, C and D were very small, with a maximum difference of 6 µm.

#### 3.4.3. Support Structure/Inclination

In the S series, all models were printed without support. For the C series, the differences between the models with and without a support structure and the SOLL model were smaller for distances A (*p* < 0.001) and D (*p* < 0.014) but larger for distances B, E (*p* < 0.002), and especially C (*p* < 0.000). In general, the deviations of all models relative to the SOLL model were <69.2 µm. In the F series, the models printed with support were closer to the SOLL model for all distances. The mean differences between the models with and without support were between 12.5 and 86.7 µm. The largest deviation from the SOLL model was for distance B for the model without support (225.6 µm). The differences in the use of support structures were significant for distances B (*p* < 0.001) and C (*p* < 0.000).

#### 3.4.4. Positions on the Build Platform

Due to the different dimensions of the build platforms and the resulting different arrangement of the models, it was not possible to compare the corresponding models between the different printers. For the S series, there were no deviations depending on the model’s position on the build platform (Figure 5), and only the differences explained in Section 3.4.1 and Section 3.4.2 were confirmed. The same could be observed for the C series (Figure 6) and the F series (Figure 7).

## 4. Discussion

Dimensional behaviour is an important factor in the production of dental restorations. Additive manufacturing processes are becoming increasingly important, both in the direct production of dental restorations and in the indirect production of supporting parts. Only sufficiently high dimensional stability—in terms of precision and accuracy—can guarantee that dental restorations fit accurately and function appropriately [23,27,41,42]. Despite the digital workflow, some work processes—for example, preparation and fit control of crowns and bridges—require working or saw-cut models as a basis [43]. In individual cases, additively manufactured models (for implant or bar restorations) also form the basis for additional production processes. According to the relevant literature, digital methods that employ scanning and matching processes are used for the measurements [5,19,24,27,36], although analogue measurements with callipers have proved successful [11,22,34,35]. In the literature, it is recommended that for prosthetic restorations, models have a deviation of no more than ±120 µm [44]. In studies, additively manufactured models have achieved an accuracy of <100 µm [10,15]. In the authors’ opinion, the upper limit of ±50 µm specified for the classic plaster models should be the aim. The mean deviations of this study were 200.9 µm for the S series, 25.2 µm for the C series, 142 µm for the F series, 72 µm for the plaster models and 74 µm for the milled models (see Table 5 and Table 6). In many studies, plaster models have demonstrated superior accuracy compared with milled or printed models [22,23,27,28,36], which is congruent with the results of the present study, with the exception of the C series.

The statistical analyses of this study showed that hypothesis (1) must be rejected: the choice of printer and material had an effect on the dimensional behaviour. Overall, compared with the C series (MWT: 25 µm), the dimensional deviation increased by a factor of 8 for the S series and by a factor of 5.6 for the F series. The differences between the individual test series may be due to the printers’ modes of operation and/or the material properties [21,45]. 

Great importance is attributed to the exposure technology. A moving exposure unit and the resulting constant distance between the light source and the object result in lower light scattering losses and thus less loss of precision due to distortion. This could not be confirmed with the highest differences for the S-series models. The light sources also vary between the printers used in the present study. On the one hand, the materials polymerise as specified by the manufacturer when different wavelengths of light are used (the S series at 385 nm and the C and F series at 405 nm). On the other hand, different modes of operation are used. While the S- and C-series printers use the DLP method, the F-series printer is based on SLA. 

The print quality is also influenced by the XY and Z resolution of the respective printers. According to the manufacturers, the greatest accuracy can be achieved with a high Z-axis resolution and the smallest possible minimum structure size (XY resolution). The Z-axis resolution, that is, the layer height, can be defined by the user. In the present study, it was set at 50 µm for better comparability among the series. The DLP printers have a defined pixel matrix in relation to the exposure area (build platform), which is also dependent on the projector. For each pixel, there is an actual XY value, which must be determined metrologically by the manufacturers. In the case of the S-series printer, a pixel size of 50 µm is specified with an indication for the variation of this size and the print volume, as well as an accuracy of ±25 µm (508 dpi resolution). The C-series printer is said to have a resolution of 1920 × 1080 with a minimum structure size of 65 µm. Analogously to the C-series values, a sufficiently high dimensional stability can be achieved with a pixel size of 65 µm. Despite the smaller minimum structure size declared by the manufacturer, the differences were larger for the S-series models. The cause could be due to the shape of the pixels: they do not have to be exclusively square but can also be diamond-shaped depending on the projector. The latter would produce different sizes in the X and Y directions and, accordingly, may have had a negative effect on precision or dimension [46]. In SLA technology, the smallest structure size in the ideal imagination is defined by the laser spot size. The smaller the value, the higher the level of detail. The laser spot is specified as 140 µm for the F-series printer. It describes the smallest movement that the laser is capable of making within a layer. Investigations by the manufacturer proved reliable XY structures only at a dimension of 150 µm, which was set as the minimum structure size [47]. Therefore, the more than doubled minimum structure size compared with the C-series printer could be a reason for the larger differences compared with the SOLL model. 

In the literature, researchers have stated that with a higher resolution or lower slice height in the Z-direction, precision can decrease [24]. The possible causes are an increase in the number of exposure layers and greater repositioning of the build platform in the Z direction, leading to an increased potential for errors, artefacts and failures during the course of a print [24,46,48]. In this context, overexposure of already-exposed layers can also play a role. Depending on the material composition and colour, the projected light can penetrate into deeper levels and cause distortion [9]. On the other hand, Zhang et al. [49] found that for SLA printing, accuracy increases as the layer thickness decreases. The authors stated that nonlinear edges in the printed object are not directly positioned on the Z or X/Y plane. Therefore, the layer thickness determines the number of discrete points. A thinner layer creates multiple discrete dots and thus a smoother and more detailed surface, making printing more accurate. In contrast, a thicker layer has fewer discrete points with wider spacing, which leads to a staircase effect at the edge and affects accuracy [49]. Because the coating thickness in the present study was just 50 µm, no statements can be made regarding possible changes within the series. Hence, this issue requires further investigation.

The S- and C-series printers are semi-closed and closed systems, respectively. Thus, the materials recommended by the manufacturer for the test specimens were used in this study. These materials were liquid photopolymers based on acrylates or methacrylates (Table 3). Consistent with the literature, the test specimens underwent shrinkage due to the polymerisation process [18]. In this process, long-distance bonds are replaced by strong and short covalent bonds between carbon atoms of different types within the monomer units via van der Waals force. The ultraviolet (UV) active monomers radicalise and convert into polymer chains [50]. As a result of the choice of printer-specific materials, the shrinkage factor of the materials may have varied independently of the printer and may have been most pronounced in the S-series models. Basically, the composition of the resin, specifically the photo-initiator concentration, in combination with the processing procedure influences the mechanical, biocompatible and aesthetic aspects of the printed components [45,51,52,53,54,55]. Studies have also shown that different exposure sources during post-polymerisation can cause property changes, for example, the breaking load of temporary plastic materials [45]. In conclusion, not all light-curing devices can activate reactive groups equally effectively, and polymerisation is affected by the irradiation intensity and duration [56]. The polymer conversion rate, the true crosslink density in the polymer network, can be influenced and varies depending on the processing. Therefore, in principle, the volumetric shrinkage of the printed components is dependent on the chemical reaction that occurs and, according to Schümann et al. [57], also on process-related thermal changes. In the present study, the test specimens were subjected to post-polymerisation. Depending on the manufacturer’s specifications and the light-curing unit, the times varied considerably (6.67 min for the S series, 10 min for the C series and 60 min for the F series), meaning that there were variable temperature influences depending on the series. Compared with the C-series models, the F-series models were exposed to a sixfold increase in time and faced with the associated heat that was generated. Depending on the coefficients of thermal expansion, it can be assumed that thermal expansion increases with temperature and that internal stresses, which could lead to geometric distortions, increase.

The manufacturers provided relatively little information on the materials used in the present study (i.e., on the safety data sheets). Thus, no specific conclusions can be drawn about the materials used. The density was only specified for the C series (1 g/cm^3^) and the F series (1.08 g/cm^3^), and the difference was relatively small. The density of the starting resins can be varied by adding particles, which can affect the liquid and solid density in the form of an increase in density. The particles can also influence the crosslinking process during printing and post-processing. In this context, the crosslinking reaction could be limited by hindering the mobility of the polymer molecules [58] or accelerated by specific surface modifications or complex interactions between the matrix and particles [59,60]. Both factors could counteract this phenomenon. The influence of the additive process itself and the post-curing process was not investigated in the present study; that endeavour would have required an intermediate measurement.

In addition to the selection of the resin, it is important that the hardware is calibrated and, for example, that the exposed pixels of the 3D printer match the *stl* file. For this purpose, the manufacturer usually provides corresponding calibration parts. The specimens were manufactured based on the company specifications or the specifications of the dental laboratory. At the time of the study, it was assumed that the printers were calibrated correctly, but no corresponding certificates were available. 

Hypothesis (2) must be partially rejected. While the exact positioning on the build platform did not produce any clearly visible changes, the orientation and alignment of the models to the front side of the respective printers showed, in part (the C and F series), distance-specific differences in the dimensional behaviour. For the C series, the measurements of the models oriented across to the front were slightly closer to the SOLL model measurements. As proposed by Lederer et al. [46], a pixel geometry deviating from the square shape could be the cause for this outcome. Each underlying micromirror can change its orientation between 1° and 12° to the beam axis, exposing and curing only the desired areas. The XY resolution is determined by the pixel size [61]. This means that the minimum structure size is distributed differently with respect to the basic shape of the test specimens and also has different sizes depending on the direction. There is no comparable study in the literature that has investigated dimensional stability that chooses the printer front as the reference point of alignment. Some researchers have investigated the build orientation, where the term perpendicular referring to the *Z*-axis—that is, the specimens were printed upright [9,62,63]. Park et al. [64] investigated the influence of the orientation on the dimensional accuracy of a bridge, where the orientation was chosen at different rotation angles around the *XY*-axis. They also concluded that depending on the orientation of the structure, the shape of the exposure surface changes, and, therefore, the shape and degree of polymerization shrinkage are affected. 

The measurements of the F-series models showed the greatest differences from the SOLL model, especially for the longest distance (B). In the F-series printer, polymerisation is activated sequentially by a laser dot. Despite the monofrequency and linearly polarised laser light, these beams are subject to divergence. This is also optimised by optical systems such as mirrors or lenses. However, beam propagation, the divergence of the beam, cannot be completely prevented and increases the longer the beam path is. In addition, when generating the laser light, transverse oscillation modes in the laser resonator make it possible to change the beam (in height and width) in different spatial directions. Depending on the arrangement of the laser in the printer and the distance of the test specimens, minimal deviations from the optimum diameter (140 µm) could add up—especially over longer distances—and be reflected in the measurements. Favero et al. [24] confirmed the influence of the laser spot and radical polymerisation kinetics on the resolution of the SLA print. Poorer XY resolution could lead to physical pressure outside the print object boundary. Kim et al. [21] noted that the SLA technique is prone to errors due to the mirror and the comparatively slow laser motion. The selection of laser intensity and speed to avoid refraction of the light are critical to the reproducibility of SLA printing [65,66]. Shim et al. [62] stated that the refraction of light in the SLA print was lower to the vertical axis than to the *XY*-axis, which in the present study might have provided better dimensional stability by arranging the samples differently along the *Z*-axis. In contrast, the faster DLP process minimises the error probability associated with repeat printing. 

Comparison of the solid and hollow models revealed section-specific differences, so hypothesis (3) must be partially rejected. The results showed that linear shrinkage can be different from 3D shrinkage. Indeed, the S-series model measurements showed a comparatively large shrinkage for distances A–D, which resulted in a positive difference compared with the master model in distance E, amplified in the hollow models. There was a similar—but significantly less pronounced—effect in the C-series hollow models. In contrast, there were consistently negative differences for the F series. Again, the hollow models showed somewhat increased shrinkage. These findings suggest that for non-uniform wall thicknesses and flat or wide parts, temperature variations are more likely to cause deviations and deformations to the desired geometry. On the contrary, researchers have reported no relevant differences and have recommended hollow structures to reduce printing time, material consumption and costs [10,11]. Chuang et al. [67] found that homogeneous shrinkage occurred on smooth, straight surfaces, which they expected based on the uniform contact with the polymer. Rungrojwittayakul et al. [10] observed asymmetric shrinkage patterns on occlusal depressions, which they attributed to a lack of direct exposure opportunity. In the present study, the hollow models had a more complex geometry, which made direct exposure of all surfaces difficult. The drain holes in the design are indispensable to avoid the accumulation of liquid resin inside the structure [68,69]. In addition to deformations due to the container effect (surface tensions), discolorations are a possible consequence. The approximately 20% larger surface area of hollow patterns increases the adhesion of more uncured resin and thus the washing effort. The larger surface area also presents the risk of more stresses during post-curing. Because of the reduced contact areas in hollow models, adhesion problems to the build platform have been observed [68].

**Figure 17:**
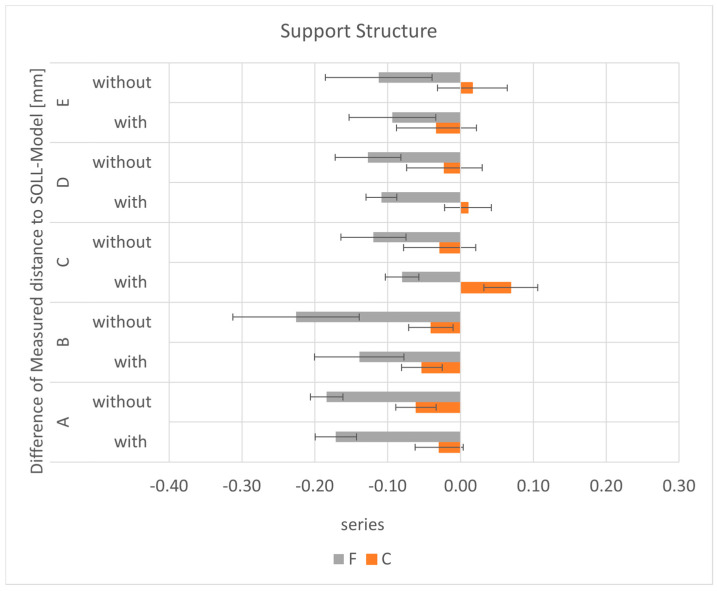
The mean differences as a function of the distances and support for each series.

**Figure 18:**
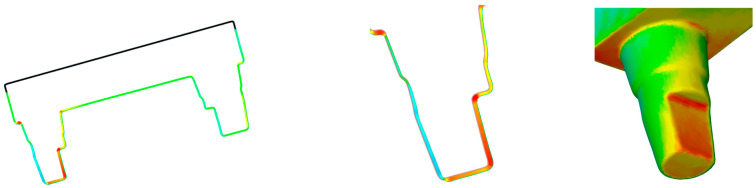
Colour map of the differences between the SOLL model and a C-series model.

There were significant differences between models with and without support structures, so hypothesis (4) must be rejected. On average, the F-series models printed with a support structure showed smaller measurements compared with the SOLL model. As already mentioned, the final shrinkage of the test specimens depends on the material, the chemical setting process and the environmental influences or temperature variations during the printing process. The shrinkage process involves physical shrinkage of the polymer during curing and the change in geometry due to the specific thermal expansion coefficient during cooling of the material, both of which lead to internal stresses. These can be amplified by constraining edge conditions, in this case by the continuous adhesion of the specimens to the build platform, and lead to larger dimensional changes compared with specimens with a support structure. Overhangs demand the use of support structures to prevent sagging and delamination of the component [69]. Alharbi et al. [70] found that the number and geometry of support structures can affect accuracy. A high number of support structures introduces potential errors when they are separated from the part. It is postulated that ideal alignment, with maximal self-supporting surfaces, can minimise defects and the time required for finishing and polishing. These points are very important, especially for directly printed dentures, and less so for bases of models. In general, the distribution of the supports had a greater influence than the diameter of the supports [70]. Unkovskiy et al. [63] reported that for samples without support, there was a significant deviation in the *Z*-axis. The cause seems to be the first layer on the build platform. To ensure a secure hold of an object, the first layer is irradiated for a longer time, which can lead to a compression and thus a shorter total height and an overhang in the width. Because the lower area was trimmed when measuring the models, no direct comparison can be made to evaluate this view, although the differences in direction A could be influenced by this phenomenon. Osman et al. observed positive deviations on printed objects in their study. They suspected that the upward movement of the build platform during the fabrication process was due to sagging of the material under its own weight in combination with the curing pattern of the DLP technique. Over-hardening or post-hardening of the coatings was indicated as the cause. For the C-series model with support, there were positive differences in distance C (Figure 17). A more detailed analysis showed that, compared with the SOLL model, there was material accumulation on the flattened side of the smaller die (Figure 18). Therefore, it is reasonable to assume that the inclined position of the flat surface of die C resulting from the placement of the support structure prevented initially uncured polymer from flowing off. Due to ambient light or curing of the subsequent layers, the residual polymer may have subsequently hardened undesirably. Given that the inclination of the models was identical in all series, this phenomenon was likely related to the material. In addition to a lower viscosity, surface modifications (charges) may be the cause of the buildup.

In the literature, it is recommended that for prosthetic restorations, models have a deviation of no more than ±120 µm [44]. This recommendation is justified by possible consequential errors that affect the accuracy of fit of the restorations—for example, if a shrunk model is produced, the restoration to be fabricated would be fitted on a model that is relatively too small and would therefore be produced too small. In the present study, most 3D-printed models achieved the level of accuracy found in the literature, although there were significant differences depending on the printer and the printing parameters used. Unfortunately, no generalised recommendations could be derived from the present results. From a technical point of view, it would therefore be highly recommended to use test prints to determine the respective ideal orientation of the components to be fabricated, e.g., splints, bridges and crowns.

The comparison of the measurement methods showed smaller differences mainly for the analogue method. Considering all of the distances, the deviation was 79 µm. Given the error variables (measurement error, scan error, alignment error during superposition), this amount is within the clinical tolerance, so hypothesis (5) can be accepted. Studies have shown that printing parameters such as layer thickness and the type and number of support structures can influence the surface quality [12]. A distinction is made between the real surface of the workpiece in relation to its environment and the actual surface. The latter is defined as the surface that can be measured and mapped, and thus reflects only the approximate image of the surface. The calliper measurements were carried out for the outer dimension (the distance from A to D) with the outer measuring legs. The inside dimension (distance E) was measured with the inside measuring legs of the calliper. For the most accurate representation of the section extent, the largest possible inner leg area was used to measure each section, although the planar area of the legs to the section to be measured on the workpiece is severely limited and far from equal to the total area of the workpiece. The measurement basis for the legs is exclusively the outermost elevations of the surface, which corresponds to the maximum roughness height (Rmax) when considering the classic surface profile. Surfaces form the basis for software measurement. A distinction is made between the geometrically ideal surface, which is usually given by the nominal value in design drawings or constructions, and the actual measurable surface. The present study did not use a purely virtually developed CAD body. According to the clinical situation—analogous to patient care—a highly simplified geometric bridge was scanned and used as the original dataset. As a result, the 3D-printed model did not have flat surfaces over the entire side, and the software specified minimum and maximum values for the actual values (Figure 13). From this, the mean value was calculated. The findings of the present study may provide an explanation for the differences between the two measurement methods.

Matching displays the root mean square, which is used extensively in the literature and corresponds to the arithmetic mean of the positive and negative deviations of the entire area. Thus, this value shows the deviation between the SOLL model and the actual model as a whole. Due to the intended comparison of the different measurement methods and the labelling of the models shown in Figure 12 and the sample cutting from the basal side, this value was not used for evaluation in the present study. According to the software programs, the alignments are often performed automatically. Figure 10 and Figure 11 clearly show that this approach may be insufficient without fine adjustment. The initial alignment of the specimens serves only as a rough preliminary alignment so that the elements constructed for further alignments can be reasonably calculated. The actual main alignment corresponds to the fine adjustment, where all mesh data are mathematically assigned to the CAD data in a deliberate way that is always the same, an approach that provides reproducibility and stability in the evaluation. 

## 5. Conclusions

Although there were differences in accuracy between the models of the various manufacturing processes in this investigation, they were all within the range of clinical acceptance described in the literature. No general manufacturing recommendation for 3D-printed models can be derived from the present results. In general, there was no influence on accuracy due to the positioning on the build platform. In contrast, the measured values reflected printer-specific differences depending on the orientation of the samples to the respective printer front. Full models and the use of support structures tended to produce higher accuracy. When selecting support structures, however, printer- and material-specific defective areas (incorrect polymerisation of residual resin that does not flow off, overhangs) may be possible. It was not possible to determine the influence of the material on accuracy due to the very limited information provided by the manufacturers. Thus, individual printer- and specimen-specific workflows are indispensable to ensure high accuracy and precision.

The comparability of the overall results of digital calliper versus 3D measurements suggests regular application of these approaches in clinical practice.

## Figures and Tables

**Table 1:** Description of the measuring distances.

Measuring Distances	Description
A	Short side of base
B	Long side of base
C	Diameter of the small stump
D	Diameter of the large stump
E	Distance between the stumps

**Table 2:** An overview of the printers and the printing materials.

Printer	SolFlex 350	CaraPrint 4.0	Form2
Manufacturer	VOCO GmbH (Cuxhaven, Germany)	Kulzer GmbH (Hanau, Germany)	Formlabs GmbH (Berlin, Germany)
Technology and exposure unit	Digital light processing (385 nm)	Digital light processing (405 nm)	Stereolithography (405 nm) Laser with 250 mW
Editing software	Autodesk Netfabb 2020	Cara Cam 1.0	PreForm 2.13.1
Build platform	64 × 120 mm Elongated Perforations	58 × 103 mm Smooth Surface	145 × 145 mm Flexible Printing Surface
Maximum construction height	130 mm	130 mm	175 mm
**Sample Series**	**S**	**C**	**F**
Models (n) per print Prints	2 × *n* = 12 S1+S2	6 × *n* = 8 C1–C6	3 × *n* = 18 F1–F3
Material	V-Print Model Beige	Dima Print Stone Beige	Grey Resin V3
Printing Parameters			
Layer thickness	50 µm	50 µm	50 µm
Inclination (support structure) 0° = without support 15° = with support	0° (*n* = 24)	0° (C1, C3, C5)/15° (C2, C4, C6) (*n* = 24/*n* = 24)	0° (F2)/15° (F1, F3) (*n* = 18/*n* = 36)
Model structure	Full (*n* = 12) Hollow (*n* = 12)	Full (*n* = 24) Hollow (*n* = 24)	Full (*n* = 27) Hollow (*n* = 27)
Position on the build platform *	1 Front left	1 Front centre	1 Front left
	2 Centre left	2 Back centre	2 Centre links
	3 Back left	3 Left centre	3 Back links
	4 Front right	4 Right centre	4 Front middle
	5 Centre right		5 Centre middle
	6 Back right		6 Back middle
			7 Front right
			8 Centre right
			9 Back right
Placement *: model base orientation	Parallel—P Across—A (*n* = 12/*n* = 12)	Parallel—P Across—A (*n* = 24/*n* = 24)	Parallel—P Across—A (*n* = 24/*n* = 30)

* Relative to the respective printer front.

**Table 4:** The values of the master model from GOM Inspect Professional software and the calliper measurements.

	A	B	C	D	E
SOLL model (GOM Inspect Professional software 2021)	8.99	36.03	3.24	4.11	24.67
Calliper	8.99	36.02	3.24	4.14	24.62

**Table 3:** The composition of the resins.

VOCO: V-Print Model Beige		
	Polyetherdimethacrylate	50% to 100%
	Tripropylenglycoldiacrylate	10% to 25%
	Hydroxypropylmethacrylate	5% to 10%
	Diphenyl (2,4,6-trimethylbenzoyl) phosphineoxide	≤1%
**Kulzer: Dima Print Stone Beige**		
	7,7,9-Trimethyl-4,13-dioxo-3,14-dioxa-5,12-diazahexadecane-1,16-diylbismethacrylate	≥25% to ≤50%
	(octahydro-4,7-methano-1H-indenyl) methylacrylate	≥10% to ≤20%
	Tris (2-hydroxyethyl) isocyanuratetriacrylate	≥10% to ≤25%
	Bisphenol-A-Polyethylenglycoldietherdimethacrylate	
	(2,4,6-trioxo-1,3,5-triazinane-1,3,5-triyl) Triethylenetriacrylate	≥10% to ≤25%
	Tricyclodecanedimethanoldiacrylate	≥5% to ≤25%
	Phenylbis (2,4,6-trimethylbenzoyl) phosphineoxide	≥1% to ≤5%
	2-Hydroxy-4-methoxybenzophenon	≥0.25 to ≤1%
**Formlabs: Grey Resin V3**		
	Methacrylated oligomer	≥75% to ≤90%
	Methacrylated monomer	≥25% to ≤50%
	Diphenyl (2,4,6-trimethylbenzoyl) phosphine oxide	≥1% to ≤3%

**Table 5:** The general results showing the differences in the deviations between the calliper and GOM Inspect Professional software measurements compared with the digital SOLL model (all measurements are in mm).

	A	B	C	D	E	∑
**S GOM**	**−0.2229**	**−0.3271**	**−0.1704**	**−0.1717**	**0.1125**	**I0.201I**
S Calliper	−0.1012	−0.2528	−0.1042	−0.0603	0.0453	I0.113I
**C GOM**	**−0.0450**	**−0.0467**	**0.0204**	−0.0058 *	**−0.0083**	**I0.025I**
C Calliper	0.0153	0.0240	*0.1178*	0.1220 *	*−0.0282*	I0.061I
**F GOM**	**−0.1794**	**−0.1967**	**−0.1063**	**−0.1207**	−0.1059 **	**I0.142I**
F Calliper	−0.1386	−0.1437	−0.0030	0.0067	−0.0857 **	I0.075I

* The largest deviation was 128 µm. ** The smallest deviation was 20 µm.

**Table 6:** The reference model measurements determined with the GOM Inspect Professional software.

Reference	A	B	C	D	E	∑
GOM plaster models	0.0760	0.0720	−0.0400	0.0220	−0.1520	I0.0724I
GOM milled models	−0.1225	−0.1700	−0.0300	−0.0325	−0.0175	I0.0745I

## Data Availability

The data presented in this study are available on request from the corresponding author due to privacy reasons.
